# Predictive Factors and Onset Timing of Delirium in Hospitalized Patients with Heart Failure

**DOI:** 10.31662/jmaj.2025-0253

**Published:** 2025-11-28

**Authors:** Noriko Kawazoe, Yoshiaki Kubota, Takuya Nishino, Miwako Ogane, Yoshiki Iwade, Daisuke Hayashi, Yukihiro Watanabe, Katsuhito Kato, Shuhei Tara, Kuniya Asai

**Affiliations:** 1 Department of Nursing, Nippon Medical School Hospital, Bunkyo-ku, Tokyo, Japan; 2 Department of Cardiovascular Medicine, Nippon Medical School, Bunkyo-ku, Tokyo, Japan; 3 Department of Health Care Administration, Nippon Medical School, Bunkyo-ku, Tokyo, Japan; 4 Department of Pharmaceutical Service, Nippon Medical School Hospital, Bunkyo-ku, Tokyo, Japan; 5 Department of Hygiene and Public Health, Nippon Medical School, Bunkyo-ku, Tokyo, Japan

**Keywords:** heart failure, delirium, total anticholinergic load

## Abstract

**Introduction:**

This study aimed to examine the predictive factors and timing of delirium onset in hospitalized patients with heart failure, focusing on the impact of total anticholinergic load and other contributing variables.

**Methods:**

The single-site retrospective cohort study included 694 patients hospitalized for heart failure and receiving treatment for hyperpolypharmacy between January 2015 and March 2023. The patients were categorized into delirium and non-delirium groups, with the delirium group further subdivided into early-onset (within 6 days) and late-onset (day 7 or later) subgroups. Logistic regression analyses were performed to identify significant factors associated with delirium onset.

**Results:**

Compared with the non-delirium group, the delirium group (n = 54) showed a higher total anticholinergic load, malnutrition prevalence, and elevated N-terminal pro-brain natriuretic peptide levels. Early-onset delirium was associated with a higher total anticholinergic load and C-reactive protein levels, whereas late-onset delirium correlated with malnutrition. Hyperactive delirium was predominant in the early-onset group and the hypoactive or mixed subtypes in the late-onset.

**Conclusions:**

Elevated anticholinergic loads and the presence of infection were primary contributors to early-onset delirium; malnutrition and the body mass index were critical for late-onset delirium. These findings emphasize the need for targeted preventive strategies based on delirium onset timing.

## Introduction

Congestive heart failure (HF) is a cardiovascular disease with a high global prevalence and mortality rate, posing significant social and economic burdens in both developed and developing countries ^(1), (2)^. With the aging population and continuous advancements in healthcare, the number of patients with HF is expected to increase further, leading to a global decline in the quality of life ^(3)^.

Delirium, characterized by abrupt changes in attention, cognition, and awareness, is often triggered by underlying conditions such as HF that cannot be explained by pre-existing neurocognitive disorders ^(4)^. Moreover, HF has been identified as an independent risk factor for delirium ^(5)^. The presence of delirium in patients with HF complicates communication with healthcare providers, increasing the challenges faced by the medical staff ^(6)^. Multiple studies have demonstrated associations between delirium and adverse outcomes in patients with HF, including higher mortality rates, prolonged hospital stays, and increased healthcare costs ^(4), (7), (8), (9), (10), (11), (12)^. Additionally, patients with HF are often subject to polypharmacy ^(3)^, which can escalate into hyperpolypharmacy (HPP), a condition also reported in this population ^(13)^. Among various medications, the total anticholinergic load (TAL) has been identified as a significant risk factor for delirium ^(14)^. However, the diagnosis of delirium in patients with HF is often delayed, and current healthcare practices fail to adequately address the needs of these patients ^(4)^. Although delirium frequently occurs in older adults and patients with chronic illnesses, potential differences in the contributing factors based on the timing of delirium onset remain unclear. Additionally, the associated risks have not been fully elucidated. We hypothesized that risk factors differ across subgroups, depending on the timing of the onset of delirium. Therefore, this study examined the impact of TAL and other factors on the timing of delirium onset in patients with HF and HPP.

## Materials and Methods

### Design

This study followed a quantitative research design.

### Population and sample

The inclusion criteria were urgent admission to our hospital for HF and treatment for HPP. Patients with urgent hospitalization included those requiring immediate HF management but excluded those under deep sedation or mechanical ventilation, which were situations where delirium assessment was impractical. HPP was defined as taking at least 10 medications at the time of admission. First, we categorized patients into two groups based on the presence or absence of delirium. The delirium group was further subdivided into two categories based on onset timing: an early-onset group (onset within 6 days of admission) and a late-onset group (onset on or after day 7 of admission). Supplementary Figure 1 presents a histogram of the delirium onset times. Early-onset delirium was defined as delirium with onset within a median of 6 days after admission. We analyzed differences in the patient backgrounds and examined factors influencing the occurrence and timing of delirium onset.

Delirium is defined in the Diagnostic and Statistical Manual of Mental Disorders, Fifth Edition (DSM-5) based on the following five key features ^(15)^:

1. Disturbance in attention and awareness.

2. Development over a short period, with fluctuations in severity throughout the day.

3. Additional disturbance in cognition.

4. These disturbances are not better explained by a pre-existing neurocognitive disorder.

5. There is evidence that the disturbance is a direct consequence of another medical condition.

Delirium was retrospectively diagnosed through a review of clinical records, including psychiatric consultations, or calculated for inpatient psychotherapy.

TAL was calculated based on the anticholinergic cognitive burden (ACB) scale developed by Boustani et al. ^(14)^. Each prescribed medication was assigned a score from 1 to 3 according to its anticholinergic potency, and the total ACB score was calculated as the sum of these individual scores. Supplementary Table 1 shows a detailed list of medications contributing to TAL. Malnutrition was defined according to a serum albumin level of <3.5 g/dL at the time of admission. New-onset atrial fibrillation was defined as atrial arrhythmias (including atrial fibrillation, atrial flutter, and atrial tachycardia) occurring prior to delirium onset.

### Exclusion criteria

The exclusion criteria were in-hospital death and use of fewer than 10 drugs at admission.

### Data sources

This single-site cohort study was based on information collected from the Diagnosis Procedure Combination (DPC) database of Nippon Medical School Hospital. The following data were extracted: patient age and sex, primary diagnoses and comorbidities, medications, and discharge status. The diagnoses followed the guidelines of the International Classification of Diseases, 10^th^ revision. Laboratory data and electrocardiograms were collected electronically from the medical records.

### Data analysis

The primary endpoints of this study were the differences in the patient backgrounds, factors influencing the occurrence of delirium, and the timing of its onset.

Categorical variables were expressed as numbers and percentages and compared among the groups using the chi-squared test. Continuous variables were expressed as means and standard deviations or medians and interquartile ranges. Logistic regression analysis was performed to identify the factors associated with delirium occurrence. Variables included in multivariable logistic regression analysis were selected according to their clinical relevance and statistical significance in univariate analyses (p < 0.05). Two-sided p values <0.05 were considered to indicate statistical significance. The statistical analyses were performed using SPSS version 28 (IBM Corp., Armonk, NY, USA) and R software (version 4.2.1; R Foundation for Statistical Computing, Vienna, Austria).

### Ethical considerations

The data were anonymized, and their use precluded the identification of individuals. Consequently, the requirement for patient consent was waived. The study was conducted in accordance with the Declaration of Helsinki and approved by the Institutional Review Board of the Nippon Medical School Hospital (B-2021-433).

## Results

### Participants

**Figure 1.**
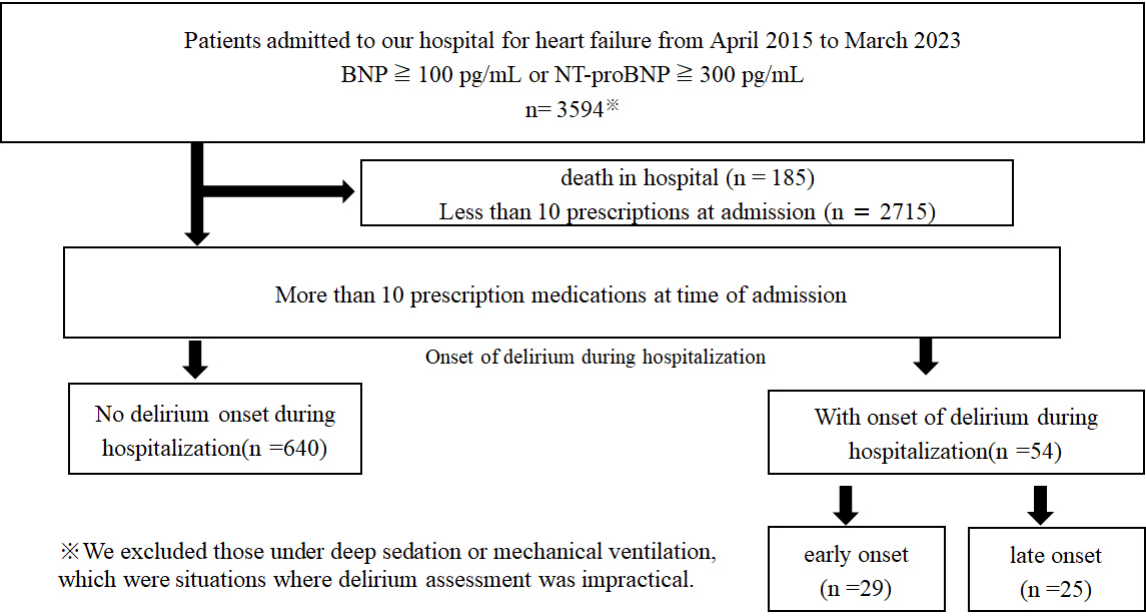
Flow chart showing the process of patient selection. Between April 2015 and March 2023, this study enrolled 694 patients with hyperpolypharmacy (taking 10 or more medications at admission) who were hospitalized at our institution for heart failure.

Between April 2015 and March 2023, this study included 694 patients with HPP (taking ≥10 medications at the time of admission) who were hospitalized at our institution for HF (Figure 1). The mean age of the cohort was 76.0 ± 11.1 years, and 64.1% were men.

The participants were divided into the delirium (n = 54) and non-delirium (n = 640) groups. Compared with the non-delirium group, the delirium group exhibited a higher total anticholinergic burden, malnutrition, incidence of infections, and elevated levels of the N-terminal pro-brain natriuretic peptide (Table 1). The total number of medications did not differ significantly between the groups. Supplementary Table 2 shows the HF-related medication profiles across the four patient groups.

**Table 1.** Background Data of Patients in the Four Groups.

Variable	Non-delirium group	Delirium group	p Value	Early-onset	Late-onset	p Value
N	640	54		29	25	
Age (years)	78 (70-84)	80 (69-85)	0.234	83 (73-85)	80 (68-85)	0.761
Men, n (%)	416 (65.0)	29 (53.7)	0.130	14 (48.3)	15 (60.0)	0.557
BMI ≥25 kg/m^2^, n (%)	193 (31.1)	20 (37.0)	0.457	10 (34.5)	10 (40.0)	0.988
LVEF (%)	57 (39-66)	54.50 (35-67)	0.494	54 (35-64)	58 (33-68)	0.979
Length of stay (days)	14 (9-22)	26 (16-40)	<0.001	18 (15-29)	30 (21-60)	0.008
Alb (g/dL)	3.7 (3.3-4.0)	3.5 (3.0-3.8)	<0.001	3.7 (3.1-3.9)	3.4 (2.9-3.6)	0.106
Alb <3.5 g/dL, n (%)	202 (31.5)	26 (48.1)	0.023	13 (44.8)	13 (52.0)	0.607
CRP (mg/dL)	0.49 (0.16-2.02)	0.88 (0.30-5.18)	0.013	0.81 (0.29-5.38)	0.96 (0.34-4.58)	0.896
Infection, n (%)	130 (20.3)	20 (37.0)	0.017	11 (37.9)	9 (36.0)	0.886
eGFR (mL/min/1.73 m^2^)	46 (29-60)	45 (23-59)	0.365	46 (27-62)	44 (23-52)	0.278
Hb (g/dL)	12.2 (10.5-13.8)	11.7 (10.8-13.2)	0.384	11.7 (10.9-12.9)	11.7 (10-13)	0.487
K (meq/L)	4.2 (3.8-4.6)	4.1 (3.9-4.6)	0.853	4.1 (3.9-4.4)	4.3 (3.9-4.6)	0.244
Na (meq/L)	139 (137-142)	138.50 (135-142)	0.421	138 (136-143)	139 (135-141)	0.375
NT-proBNP (pg/mL)	2,256 (874-5,345)	3,747 (2,388-8,437)	0.002	3,435 (2,590-5,887)	4,903 (1,959-8,849)	0.249
Number of medications	11 (10-13)	12 (11-13)	0.245	12 (11-13)	12 (10-13)	0.698
TAL	1 (0-3)	2 (1-6)	<0.001	4 (2-8)	1 (0-2)	0.001
New-onset atrial arrhythmias, n (%)	24 (3.8)	3 (5.6)	0.460	2 (6.9)	1 (4.0)	1.000

Alb: albumin; BMI: body mass index; CRP: C-reactive protein; eGFR: estimated glomerular filtration rate; HFrEF: heart failure with reduced ejection fraction; LVEF: left ventricular ejection fraction; NT-proBNP: N-terminal pro-brain natriuretic peptide; TAL: total anticholinergic load. Continuous data are presented as median (interquartile ranges). Categorical data are presented as *n* (%). BMI values are presented as mean ± standard deviation. New-onset atrial fibrillation was defined as atrial arrhythmias (including atrial fibrillation, atrial flutter, and atrial tachycardia) occurring prior to delirium onset.

### Comparison between the early-onset and late-onset delirium groups

The delirium group was further subdivided based on the timing of onset into the early-onset (within 6 days of admission, n = 29) and late-onset (7 days or later, n = 25) subgroups. There were no significant differences in age, sex, or presence of anemia between the subgroups. However, TAL was significantly higher in the early-onset subgroup, whereas the hospital stays were longer in the late-onset subgroup.

Logistic regression analysis was performed to identify factors associated with delirium onset in each subgroup. In the early-onset subgroup, both univariate and multivariate analyses identified high TAL scores and elevated C-reactive protein (CRP) levels as significant contributing factors. In the late-onset subgroup, both univariate and multivariate analyses identified malnutrition as a significant contributing factor (Table 2).

**Table 2.** Logistic Regression Analysis of Early- and Late-Onset Subgroup Data.

Variable	Univariate analysis				Multivariate analysis			
	OR	CI_Lower	CI_Upper	p Value	OR	CI_Lower	CI_Upper	p Value
Early-onset subgroup								
TAL	1.254	1.159	1.360	<0.001	1.255	1.160	1.363	<0.001
Age	1.013	0.980	1.051	0.470				
Men	0.503	0.236	1.065	0.071				
BMI ≥25 kg/m^2^	1.164	0.400	1.380	0.704				
HFrEF	1.813	0.813	3.874	0.131				
NT-proBNP	1.000	1.000	1.000	0.950				
Alb	0.577	0.294	1.141	0.111				
CRP	1.054	0.996	1.106	0.043	1.064	1.000	1.121	0.029
Hb	0.986	0.837	1.162	0.867				
K	0.755	0.416	1.290	0.335				
Na	0.999	0.919	1.094	0.989				
eGFR	0.999	0.983	1.014	0.875				
ACE-I or ARB	0.875	0.412	1.943	0.733				
Beta blocker	0.799	0.378	1.719	0.558				
MRA	1.503	0.676	3.201	0.299				
SGLT2-i	0.519	0.083	1.775	0.376				
Loop diuretic	1.285	0.564	2.759	0.531				
Late-onset subgroup								
TAL	0.946	0.772	1.100	0.531				
Age	1.012	0.976	1.054	0.531				
Men	0.808	0.361	1.885	0.608				
BMI ≥25 kg/m^2^	1.475	0.417	1.931	0.352				
HFrEF	1.669	0.693	3.782	0.231				
NT-proBNP	1.000	1.000	1.000	0.583				
Alb	0.266	0.127	0.547	<0.001	0.222	0.101	0.473	<0.001
CRP	1.051	0.988	1.106	0.073				
Hb	0.928	0.779	1.104	0.398				
K	1.325	0.785	2.086	0.257				
Na	0.930	0.856	1.015	0.091				
eGFR	0.986	0.969	1.003	0.126				
ACE-I or ARB	0.494	0.219	1.106	0.084				
Beta blocker	1.380	0.604	3.427	0.460				
MRA	0.777	0.279	1.87	0.596				
SGLT2-i	0.955	0.222	2.835	0.941				
Loop diuretic	1.627	0.696	3.649	0.243				

ACE-I: angiotensin-converting enzyme inhibitor; Alb: albumin; ARB: angiotensin receptor blocker; CI: confidence interval; BMI: body mass index; CRP: C-reactive protein; eGFR: estimated glomerular filtration rate; HFrEF: heart failure with reduced ejection fraction; LVEF: left ventricular ejection fraction; MRA: mineralocorticoid receptor antagonist; NT-proBNP: N-terminal pro-brain natriuretic peptide; OR: odds ratio; TAL: total anticholinergic load; SGLT2-i: sodium-glucose cotransporter-2 inhibitor.

### Subtypes of delirium

The prevalence of delirium subtypes in the early- and late-onset subgroups was determined through a chart review. Subtype classification was based on the DSM-5 criteria.

In the early-onset subgroup, the distribution was as follows: hyperactive subtype, 25/29 (86.2%) patients; mixed subtype, 3/29 (10.3%); and hypoactive subtype, 1/29 (3.5%). In the late-onset subgroup, the hypoactive and mixed subtypes were observed in 5/25 (20%) patients. Although the initial presentation in the late-onset subgroup was predominantly hyperactive delirium, 7/25 (28%) patients transitioned to the mixed subtype over time, with a tendency for prolonged recovery from delirium (Supplementary Table 3).

## Discussion

This study identified high TAL, infection, and malnutrition as factors associated with delirium onset. Further analysis based on the timing of delirium onset revealed that early-onset delirium was directly influenced by an elevated TAL and infection, whereas late-onset delirium was primarily associated with malnutrition. These results underscore the need to adapt nursing care strategies according to the timing of hospitalization.

Specifically, for early-onset delirium, prehospital factors, such as TAL and the presence of infection, necessitate careful adjustment of medications to reduce the anticholinergic load and establishment of stringent measures to control medical conditions. In contrast, prevention of late-onset delirium requires targeted interventions, including rehabilitation and nutritional support, to address malnutrition.

### Factors associated with early-onset delirium

High TAL and elevated CRP levels were identified as factors associated with early-onset delirium. These factors independently affected the onset of delirium, making it a particularly challenging condition. The fact that the occurrence was noted early, after hospital admission, suggests that approaches initiated after admission may not be sufficient to prevent onset. Collaboration with pharmacists to reduce TAL in daily practice may help prevent delirium. Additionally, assessing patients’ self-care capabilities, supporting behaviors that reduce infection risk, and coordinating social resources for patients with diminished functional abilities could contribute to prevention.

Recent studies have highlighted the detrimental effects of polypharmacy on patients with cardiovascular diseases. Kanai et al. ^(16)^ reported that HPP, particularly that involving non-cardiovascular medications, was associated with increased mortality and rehospitalization rates among older patients following acute decompensated HF (CURE-HF registry). Similarly, Yamamoto et al. ^(17)^ demonstrated that polypharmacy contributed to a higher risk of major bleeding after percutaneous coronary intervention, independent of antithrombotic therapy. Although polypharmacy itself is a known risk factor for adverse outcomes, our recent study reported that the focus should be on the quality, not the quantity, of drugs ^(18)^. These findings underscore the importance of optimizing medications in the management of patients vulnerable to delirium.

### Factors associated with late-onset delirium

Low nutritional status was identified as factors associated with late-onset delirium. Although these are the predisposing factors for developing delirium, they are less likely to cause the condition independently. HF has been established as an independent risk factor for delirium ^(8), (19)^. Smith et al. reported that patients with delirium had longer hospital stays than did those without ^(19)^. Early identification of delirium risk is particularly important from a prognostic perspective ^(8)^. Patients presenting with the factors identified in this study should be classified as having a high risk for delirium, warranting systematic nursing care and multidisciplinary interventions involving dietitians, physical therapists, and other healthcare professionals. Delirium has been observed in both young and older patients and is associated with adverse outcomes within 30 days ^(20)^. These findings highlight the critical importance of evaluating and managing delirium in patients with acute HF regardless of age.

### Strengths and limitations

The study was conducted on delirium that developed in patients with HF admitted to an advanced medical center after preventive measures were taken. This study has some limitations. First, the single-center DPC database design may have introduced a selection bias. Specifically, there was a possibility of immortal time bias, because patients discharged before the development of delirium could have been misclassified as non-delirious. Similarly, the exclusion of in-hospital deaths may have biased the results. Second, delirium was diagnosed by individual psychiatrists based on the DSM-5 criteria. Third, there is a lack of standardization in the methods for delirium assessment across studies, making it challenging to quantify potential relationships between delirium severity and outcomes in patients with HF. Several validated tools, including the Confusion Assessment Method, Memorial Delirium Assessment Scale, and Delirium Rating Scale R-98, exist to assess delirium severity ^(21), (22)^. However, the use of these tools was not explored in this study. Fourth, the study did not assess the most common causes of delirium, specifically, duration of bed rest, indwelling urinary catheterization, continuous intravenous infusion, fasting, and oxygenation. Fifth, there is no standardized assessment tool for delirium, and it is possible that delirium is underestimated for patients. Sixth, malnutrition was defined on the basis of serum albumin levels of <3.5 g/dL at admission. However, we acknowledge that albumin is influenced by inflammation and may not fully represent the nutritional status, and that no validated nutritional assessment tool was used.

### Conclusions

This study identified high TAL and malnutrition as factors associated with delirium onset. In the early-onset group, specific triggers―a high TAL and infections―were identified as the primary risk factors, whereas malnutrition was associated with late-onset delirium. These findings highlight the importance of implementing preventive care strategies tailored to the timing of delirium onset. To enhance the generalizability and robustness of our results, future studies conducted across multiple centers with larger sample sizes and harmonized assessment protocols are recommended.

## Article Information

### Acknowledgments

The authors extend their sincere thanks to all the professionals involved in patient care, including the emergency staff, technicians, medical engineers, nurses, pharmacists, physicians, and surgeons at the Nippon Medical School Hospital. This research received no specific grant from any funding agency in the public, commercial, or not-for-profit sectors.

### Author Contributions

NK conceived the study, and YK designed the study. NK and TN performed data collection, and TN conducted statistical analyses. MO, YI, DH, and YK drafted the original manuscript. All authors contributed to the final version of the manuscript for submission.

### Conflicts of Interest

None

This study was conducted after obtaining permission from the Ethics Committee of the Nippon Medical School Hospital (B-2021-433).

## Supplement

Supplementary Material

## References

[1] Alberto RM, Domingo R, Aitor A, et al. Long-term prognostic value of functional status and delirium in emergency patients with decompensated heart failure. Eur Geriatr Med. 2018;9(4):515-22. 34674495 10.1007/s41999-018-0072-0

[2] Ayatollahi Y, Liu X, Namazi A, et al. Early readmission risk identification for hospitalized older adults with decompensated heart failure. Res Gerontol Nurs. 2018;11(4):190-7. 29634848 10.3928/19404921-20180322-01

[3] Battle DE. Diagnostic and Statistical Manual of Mental Disorders (DSM). CoDAS. 2013;25(2):191-2. 24413388 10.1590/s2317-17822013000200017

[4] Beezer J, Al Hatrushi M, Husband A, et al. Polypharmacy definition and prevalence in heart failure: a systematic review. Heart Fail Rev. 2022;27(2):465-92. 34213753 10.1007/s10741-021-10135-4 PMC8250543

[5] Correale M, Altamura M, Carnevale R, et al. Delirium in heart failure. Heart Fail Rev. 2020;25(5):713-23. 31377979 10.1007/s10741-019-09842-w

[6] Han JH, McNaughton CD, Stubblefield WB, et al. Delirium and its association with short-term outcomes in younger and older patients with acute heart failure. PLOS One. 2022;17(7):e0270889. 35881580 10.1371/journal.pone.0270889 PMC9321444

[7] Heidenreich PA, Bozkurt B, Aguilar D, et al. 2022 AHA/ACC/HFSA guideline for the management of heart failure: executive summary: a report of the American College of Cardiology/American Heart Association joint committee on clinical practice guidelines. Circulation. 2022;145(18):e876-94. 35363500 10.1161/CIR.0000000000001062

[8] Hutt E, Frederickson E, Ecord M, et al. Associations among processes and outcomes of care for Medicare nursing home residents with acute heart failure. J Am Med Dir Assoc. 2003;4(4):195-9. 12837140 10.1097/01.JAM.0000073964.19754.C0

[9] Inouye SK. Delirium in older persons. N Engl J Med. 2006;354(11):1157-65. 16540616 10.1056/NEJMra052321

[10] Iwata E, Kondo T, Kato T, et al. Prognostic value of delirium in patients with acute heart failure in the Intensive Care Unit. Can J Cardiol. 2020;36(10):1649-57. 32615071 10.1016/j.cjca.2020.01.006

[11] Lafo J, Singh M, Jiang L, et al. Outcomes in heart failure patients discharged to skilled nursing facilities with delirium. ESC Heart Fail. 2022;9(3):1891-900. 35293145 10.1002/ehf2.13895 PMC9065834

[12] Ni H, Xu J. Recent trends in heart failure-related mortality: United States, 2000-2014. NCHS Data Brief. 2015;(231):1-8. 26727546

[13] Pak M, Hara M, Miura S, et al. Delirium is associated with high mortality in older adult patients with acute decompensated heart failure. BMC Geriatr. 2020;20(1):524. 33272204 10.1186/s12877-020-01928-7 PMC7713169

[14] Boustani M, Campbell N, Munger S, et al. Impact of anticholinergics on the aging brain: a review and practical application. Aging Health. 2008;4(3):311-20.

[15] Ritchie C, Walters RW, Ramaswamy S, et al. Impact of delirium on mortality in patients hospitalized for heart failure. Int J Psychiatry Med. 2022;57(3):212-25. 34176306 10.1177/00912174211028019

[16] Kanai M, Minamisawa M, Motoki H, et al. Prognostic impact of hyperpolypharmacy due to noncardiovascular medications in patients after acute decompensated heart failure - insights from the clue of risk stratification in the elderly patients with heart failure (CURE-HF) registry. Circ J. 2023;88(1):33-42. 37544741 10.1253/circj.CJ-22-0712

[17] Yamamoto K, Morimoto T, Natsuaki M, et al. Polypharmacy and bleeding outcomes after percutaneous coronary intervention. Circ J. 2024;88(6):888-99. 37722886 10.1253/circj.CJ-23-0558

[18] Hayashi D, Kubota Y, Nishino T, et al. Impact of polypharmacy on 3-year mortality in patients with heart failure: a retrospective study. J Pharm Health Care Sci. 2024;10(1):34. 38956739 10.1186/s40780-024-00357-7 PMC11221177

[19] Smith MJ, Breitbart WS, Platt MM. A critique of instruments and methods to detect, diagnose, and rate delirium. J Pain Symptom Manag. 1995;10(1):35-77. 10.1016/0885-3924(94)00066-T 7714346

[20] Uthamalingam S, Gurm GS, Daley M, et al. Usefulness of acute delirium as a predictor of adverse outcomes in patients >65 years of age with acute decompensated heart failure. Am J Cardiol. 2011;108(3):402-8. 21757045 10.1016/j.amjcard.2011.03.059

[21] Wilson JE, Mart MF, Cunningham C, et al. Delirium. Nat Rev Dis Primers. 2020;6(1):90. 33184265 10.1038/s41572-020-00223-4 PMC9012267

[22] Wong CY, Chaudhry SI, Desai MM, et al. Trends in comorbidity, disability, and polypharmacy in heart failure. Am J Med. 2011;124(2):136-43. 21295193 10.1016/j.amjmed.2010.08.017 PMC3237399

