# Supplementary material for: Predictive Factors and Onset Timing of Delirium in Hospitalized Patients with Heart Failure
Source: JMA J. 2025 Nov 28;9(1):254–60. doi: 10.31662/jmaj.2025-0253 (PMC12888989; doi:10.31662/jmaj.2025-0253)
Supplement: Supplementary Material [file 2433-3298-9-1-0254-s001.pdf]

Supplementary Figure 1. Histogram showing the delirium onset times

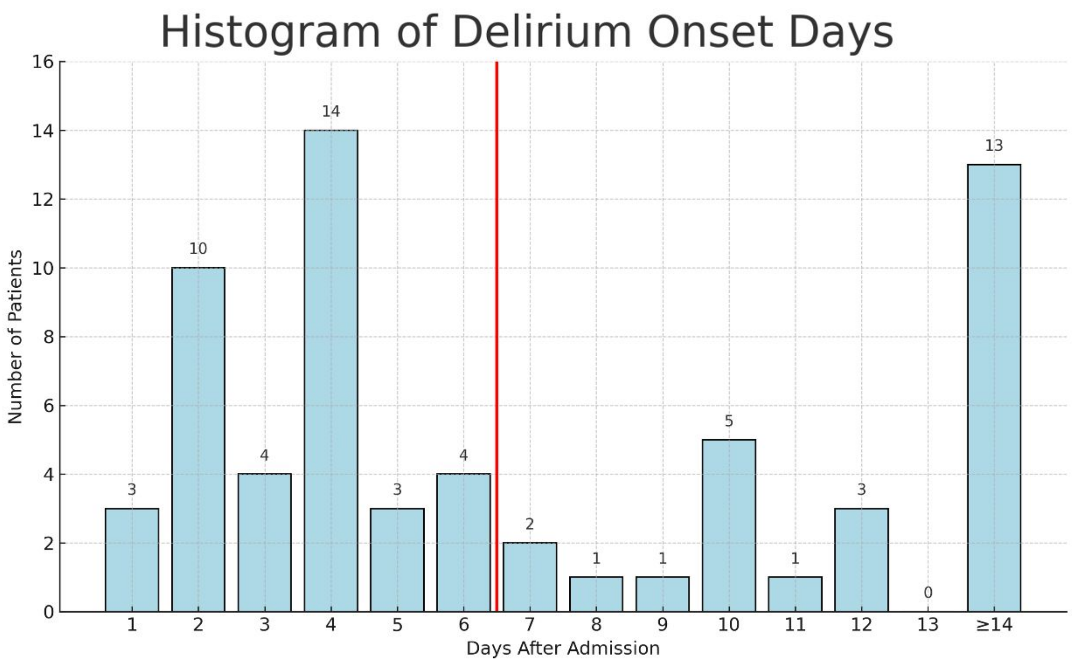

Supplementary Table 1. List of medications contributing to the total anticholinergic load

Variable	Non-delirium group	Delirium group	P value	Early-onset	Late-onset	P value
N	640	54		29	25	
H2 blockers, n (%)	41 (6.4)	4 (7.4)	0.771	3 (10.3)	1 (4.0)	0.615
Prednisolone, n (%)	62 (9.7)	4 (7.4)	0.809	3 (10.3)	1 (4.0)	0.615
Antidiabetic drug, n (%)	206 (32.2)	14 (25.9)	0.366	7 (24.1)	7 (28.0)	0.766
α-blocker, n (%)	32 (5.0)	1 (1.9)	0.504	1 (3.4)	0 (0)	1.000
Digoxin, n (%)	43 (6.7)	2 (3.7)	0.567	1 (3.4)	1 (4.0)	1.000
Psychotropics, n (%)	35 (5.5)	4 (7.4)	0.534	3 (10.3)	1 (4.0)	0.615
Benzodiazepines, n (%)	176 (27.5)	21 (38.9)	0.084	14 (48.3)	7 (28.0)	0.166
Non-benzodiazepines, n (%)	113 (17.7)	11 (20.4)	0.582	5 (17.2)	6 (24.0)	0.736
Non-dihydropyridine CCB, n (%)	40 (6.2)	1 (1.9)	0.359	1 (3.4)	0 (0)	1.000
NSAIDs, n (%)	51 (8.0)	6 (11.1)	0.435	2 (6.9)	4 (16.0)	0.399

Abbreviations: CCB, calcium channel blocker; NSAIDs, non-steroidal anti-inflammatory drugs

Supplementary Table 2. Heart failure-related medication profiles across the four patient groups

Variable	Non-delirium group	Delirium group	P value	Early-onset	Late-onset	P value
N	640	54		29	25	
ACE-I or ARB, n (%)	417 (65.2)	30 (55.6)	0.183	18 (62.1)	12 (48.0)	0.411
Beta blockers, n (%)	388 (60.6)	33 (61.1)	1.000	16 (55.2)	17 (68.0)	0.407
MRAs, n (%)	185 (28.9)	17 (31.5)	0.755	11 (37.9)	6 (24.0)	0.380
SGLT2-i, n (%)	80 (12.5)	5 (9.3)	0.665	2 (6.9)	3 (12.0)	0.653
Loop diuretic, n (%)	191 (29.8)	15 (27.8)	0.877	8 (27.6)	7 (28.0)	1.000
Statins, n (%)	425 (66.4)	36 (66.7)	1.000	22 (75.9)	14 (56.0)	0.154

Abbreviations: ACE-I, angiotensin-converting enzyme inhibitor; ARB, angiotensin receptor blocker; MRA, mineralocorticoid receptor antagonist; SGLT2-i, sodium-glucose cotransporter-2 inhibitor

Supplementary Table 3. Delirium subtypes observed in the early- and late-onset groups

Group	Hypoactive (%)	Hyperactive (%)	Mixed (%)
Early-onset delirium	5	80	15
Late-onset delirium	26	11	63
